# Overlapping Sjogren’s syndrome reduces the probability of reaching target in rheumatoid arthritis patients: a propensity score matched real-world cohort from 2009 to 2019

**DOI:** 10.1186/s13075-020-02189-w

**Published:** 2020-05-01

**Authors:** Huijuan Zhang, Haoze Zhang, Dai Gao, Wenhui Xie, Yan Geng, Zhuoli Zhang

**Affiliations:** Rheumatology and Clinical Immunology Department, Peking University First Hospital, Beijing, 100034 China

**Keywords:** Rheumatoid arthritis, Overlapping Sjogren’s syndrome, Disease activity, Therapeutic response, Propensity score matching

## Abstract

**Background:**

Overlapping Sjogren’s syndrome (SS) is not uncommon in rheumatoid arthritis (RA) and considered as a probable detrimental factor of RA. But data on the impact of overlapping SS on RA therapeutic response is limited. Our current study aimed to identify the effect in a real-world cohort from 2009 to 2019.

**Methods:**

The medical records of RA patients who visited the rheumatology clinic of our medical center from 2009 to 2019 were reviewed. Their composite disease activity scores at each follow-up point were collected. The therapeutic response between RA patients with SS (RA-SS) and without (RA-noSS) was compared. To correct confounders which may affect the therapeutic response, both propensity score matched and unmatched cohorts were analyzed by using the Cox proportional hazards model.

**Results:**

Among the 1099 RA patients, 129 (11.7%) overlapped with SS were validated by positive anti-SSA or a minor salivary gland biopsy with histological changes suggestive of SS. After propensity score matching based on their baseline characteristics, 126 of 129 RA-SS and 126 of 970 RA-noSS patients were statistically extracted. Overlapping SS was associated with a 29%, 26%, 18%, and 22% lower probability of reaching remission defined by DAS28-ESR, DAS28-CRP, SDAI, and CDAI in RA patients, respectively. Similar decreased probability of reaching low disease activity was also observed. Although ESR was most significantly affected (HR 0.69, 95% CI 0.61–0.79), other component of composite RA disease activity score was also affected by overlapping SS. Stratification by age, RF/ACPA status, or baseline DAS28-CRP was not associated with change of results.

**Conclusions:**

Overlapping SS is associated with lower probability of reaching remission or low disease activity in RA patients and should be regarded as one of the poor prognostic factors.

## Background

Rheumatoid arthritis (RA) is a chronic, inflammatory joint disease with a worldwide prevalence of about 5 per 1000 adults [1]. As one of the diverse extra-articular manifestations, sicca symptoms can be seen in 11.4 to 60.7% patients with RA [2]. Sjogren’s syndrome (SS) is a multisystem autoimmune disease characterized by hypofunction of salivary and lacrimal glands and possible systemic multiorgan manifestations [3]. SS could coexist with various connective tissue diseases, of which the incidence overlapping with RA (RA-SS) in different parts of world ranged from 3.8 to 38.7% [2, 4–12].

A growing number of cross-sectional studies have shown that RA-SS patients tend to have more severe arthritis and visceral involvement than those without (RA-noSS) [2, 11, 13]. Could the worse arthritis be attributed to a continuous poor therapeutic response? Will strengthening the management of overlapping SS reverse the poor outcome? The data regarding the impact of overlapping SS on RA therapeutic response, to our best knowledge, are scarce.

2010 witnessed the formal publication of treat-to-target (T2T) recommendation, and the T2T approach has been proved to yield superior outcomes to standard care in RA [14, 15]. Besides, there are many factors influencing the response to RA treatment including age, gender, RA duration, rheumatoid factor (RF) and anti-citrullinated peptide antibody (ACPA) status, and baseline disease activity [16, 17]. To explore the effect of overlapping SS on the therapeutic response in RA patients, we used propensity score matching (PSM) to correct those possible confounding factors.

## Methods

### Study subjects

Our current study was conducted in a tertiary university hospital. Adult patients (≥ 18 years) who met the 1987 American College of Rheumatology (ACR) criteria or 2010 ACR/European League Against Rheumatism (EULAR) classification criteria for RA were consecutively enrolled [18, 19]. The medical records from January 1, 2009, to May 31, 2019, were reviewed by two rheumatologists for definite RA and SS diagnosis based on the aforementioned criteria for RA and 2016 ACR/EULAR classification criteria for SS [3]. Any inconsistency will be decided by a third rheumatologist. The 2016 ACR/EULAR classification criteria for SS were adopted to validate the overlapping SS. In order to meet the criteria with a total score ≥ 4, either positive anti-SSA or a minor salivary gland biopsy with histological changes suggestive of SS was required.

Patients with other definite connective tissue diseases than SS were excluded from the study. Data at 5 visiting points from 4 RA patients with definite concurrent upper respiratory tract infection were discarded due to the consideration of interference of acute infection to RA disease activity assessment.

Data on demographic features and clinical and laboratory findings were retrospectively collected. The study was approved by the Institutional Research Ethics Committee, and all patients received informed consent for data collection from their medical records.

### The propensity score matched cohorts

Application of T2T concept is the key to successful RA management. The T2T concept has been generally adopted in our medical center since 2011, causing a dramatic decline in disease activity [20]. Therefore, we divided the treatment strategy into T2T and non-T2T according to the year of first visit prior to or after 2011, and “whether under the T2T concept” was used as an indicator variable to control the confounding effect caused by treatment differences. To correct other confounders which may affect RA therapeutic response in this observational study, such as age, gender, RA duration, RF and ACPA status, and baseline disease activity, DAS28-CRP in particular, we included the aforementioned variables in the logistic regression model of PSM for analysis. For each patient with RA-SS, we identified one propensity score matched RA-noSS patient using a 1:1 greedy matching algorithm [21].

### Statistical analysis

The outcome of interest was remission or low disease activity (LDA) defined by different composite disease activity indices, including Disease Activity Score based on 28-Joint Counts (DAS28), Simplified Disease Activity Index (SDAI), Clinical Disease Activity Index (CDAI), and the individual components such as 28-swollen joint count (28SJC), 28-tender joint count (28TJC), patient’s global assessment of disease activity (PtGA) on a 10-cm visual analog scale (VAS), evaluator’s global assessment of disease activity (EGA) on a 10-cm VAS, erythrocyte sedimentation rate (ESR), and C-reactive protein (CRP) [14]. Considering that remission or LDA could be achieved or lost more than once during follow-up, the Cox proportional hazard model with multiple-failure data was adopted. To ensure the robustness of the results, we performed Cox regression analysis in both matched and unmatched cohorts.

As for sensitivity analysis, we conducted subgroup analyses stratified by age (≤ 40 years, 41–60 years, ≥ 61 years), RF and ACPA status (++, +−, −+, −−), RA duration (< 6 months, ≥ 6 months), and baseline DAS28-CRP (< 2.6, 2.6–3.2, 3.2–5.1, ≥ 5.1). Furthermore, we repeated the same regression in the cohort with either trimmed at the 5th–95th percentiles of the PSM. Besides, repeated verifications in samples before deletion of data at the aforementioned infection episodes were also performed.

The results of Cox regressions were presented as hazard ratios (HRs) with 95% confidence intervals (CIs). *P* values were set two-sided with 0.05 or less considered statistically significant. All statistical analyses were performed using Stata version 14.0 (StataCorp, College Station, TX, USA).

## Results

A total of 1099 eligible RA patients were enrolled in the study, of which 129 (11.7%) overlapped with SS. Among the 129 RA-SS patients, 100 cases of overlapping SS were validated by the presence of anti-SSA and 29 by the positive histological changes of minor salivary gland biopsy supporting SS (Table S1). There were only 3 (2.3%) patients with RF but without ACPA, and all the 3 patients were classified as RA overlapping SS due to bone erosion revealed by ultrasound. In the unmatched full sample cohort, the median follow-up time was 19 (interquartile ranges, IQR 8–37) months. One hundred percent of RA-SS patients were female. One hundred sixteen (89.9%) RA-SS patients were RF positive, and 117 (90.7%) were ACPA positive, with the median age, RA duration, and DAS28-CRP at the first visit of 51 (IQR 45–61) years, 24 (IQR 8–120) months, and 3.86 (IQR 2.78–4.74), respectively, corresponding to those of RA-noSS patients with 75.6%, 86.7%, 55 (IQR 46–64) years, 24 (6–84) months, and 3.81 (2.9–4.91) (Table 1, Table S1).

**Table 1:** Critical characteristics in the unmatched and propensity score matched cohorts

	Unmatched cohort		Matched cohort	
	RA with SS	RA without SS	RA with SS	RA without SS
	(*n* = 129)	(*n* = 970)	(*n* = 126)	(*n* = 126)
Female, *n* (%)	129 (100)	733 (75.6)	126 (100)	126 (100)
Seropositive, *n* (%)	124 (96.1)	841 (86.7)	123 (97.6)	123 (97.6)
T2T, *n* (%)	107 (82.9)	851 (87.7)	105 (83.3)	112 (88.9)
Age, median (IQR) years	51 (45–61)	55 (46–64)	51 (44–61)	58 (47–64)
RA duration, median (IQR) months	24 (8–120)	24 (6–84)	24 (7–120)	12 (4–60)
DAS28-CRP at 1st visit, median (IQR)	3.86 (2.78–4.74)	3.81 (2.9–4.91)	3.86 (2.78–4.74)	3.66 (2.68–4.92)

Values are presented as *n* (%) for binary variables or median (IQR) for continuous variables. *Seropositive* positive for RF or ACPA, *T2T* treat-to-target approach, *IQR* interquartile ranges

After propensity score matching based on the aforementioned critical characteristics, 126 of 129 RA-SS and 126 of 970 RA-noSS patients were statistically extracted. The critical characteristics, RF/ACPA status in particular, were well balanced between the two groups after PSM. In the matched cohort, RA-SS patients were at moderate disease activity (DAS28-CRP 3.86 (IQR 2.78–4.74) at the first visit), with a median age of 51 (IQR 44–61) years and RA duration of 24 (IQR 7–120) months (Table 1).

The HRs for probability of reaching remission defined by DAS28-ESR, DAS28-CRP, SDAI, and CDAI with overlapping SS were 0.68 (95% CI 0.62, 0.75), 0.80 (95% CI 0.74, 0.87), 0.82 (95% CI 0.74, 0.91), and 0.77 (95% CI 0.70, 0.86) for the unmatched cohort, while in the matched cohort which critical characteristics have been corrected, the HR values remained 0.71 (95% CI 0.62–0.82), 0.74 (95% CI 0.66–0.83), 0.82 (95% CI 0.70–0.94), and 0.78 (95% CI 0.67–0.91), respectively (Table 2). A similar trend was observed when probability of reaching remission or LDA was taken as the primary outcome.

**Table 2:** Hazard ratios for reaching remission/low disease activity and individual components in RA patients associated with overlapping SS

	Unmatched cohort (*n* = 1099)	Matched cohort (*n* = 252)	Trimmed cohort (*n* = 242)
Remission based on composite disease activity score			
DAS28-ESR	0.68 (0.62, 0.75)	0.71 (0.62, 0.82)	0.74 (0.64, 0.85)
DAS28-CRP	0.80 (0.74, 0.87)	0.74 (0.66, 0.83)	0.74 (0.66, 0.83)
SDAI	0.82 (0.74, 0.91)	0.82 (0.70, 0.94)	0.83 (0.72, 0.97)
CDAI	0.77 (0.70, 0.86)	0.78 (0.67, 0.91)	0.78 (0.67, 0.91)
Boolean	0.83 (0.75, 0.92)	0.80 (0.69, 0.93)	0.82 (0.70, 0.95)
Remission/LDA based on composite disease activity score			
DAS28-ESR	0.76 (0.70, 0.82)	0.73 (0.65, 0.82)	0.74 (0.66, 0.83)
DAS28-CRP	0.80 (0.74, 0.86)	0.76 (0.68, 0.84)	0.75 (0.68, 0.84)
SDAI	0.79 (0.73, 0.85)	0.74 (0.66, 0.82)	0.74 (0.66, 0.82)
CDAI	0.78 (0.73, 0.84)	0.74 (0.66, 0.82)	0.74 (0.66, 0.82)
Individual components of disease activity score			
28SJC ≤ 1	0.83 (0.77, 0.89)	0.77 (0.69, 0.85)	0.76 (0.68, 0.84)
28TJC ≤ 1	0.81 (0.75, 0.87)	0.79 (0.70, 0.88)	0.78 (0.70, 0.88)
PtGA ≤ 1	0.81 (0.74, 0.89)	0.82 (0.71, 0.94)	0.82 (0.72, 0.95)
EGA ≤ 1	0.81 (0.74, 0.88)	0.78 (0.69, 0.88)	0.79 (0.70, 0.89)
ESR ≤ ULN	0.66 (0.61, 0.73)	0.69 (0.61, 0.79)	0.74 (0.65, 0.84)
CRP ≤ 1 mg/dL	0.84 (0.78, 0.90)	0.76 (0.68, 0.84)	0.77 (0.69, 0.85)

Values are presented as hazard ratio (95% CI) for reaching remission and/or low disease activity based on DAS28-ESR, DAS28-CRP, SDAI, CDAI, and main components in RA patients. Unmatched cohort refers to whole sample (*n* = 1099); matched cohort refers to propensity score matched (PSM) patients (*n* = 252) after correcting gender, age, RA duration, RF/ACPA status, DAS28-CRP at 1st visit, and T2T or not; trimmed cohort refers to cohort with either trimmed at the 5th–95th percentiles of the PSM (*n* = 242). ULN of ESR refers to 15 mm/h for male and 20 mm/h for female

Of note, although overlapping SS most significantly impeded the normalization of ESR (HR 0.69 (95% CI 0.61–0.79)), other individual component of composite RA disease activity score was also implicated in the poor therapeutic response of RA (Table 2).

### Sensitivity analyses

The effect of overlapping SS on the RA therapeutic response persisted when the same test was repeated in the trimmed PS-matched cohort (Table 2) as well as the intact cohort without deletion of the five data at infection episodes (Table S2). Furthermore, the detrimental effect of overlapping SS on RA therapeutic response was consistent in different subgroups of patients, regardless of stratification by age, RF and/or ACPA status, and DAS28-CRP at first visit (Table 3, Table S3). The 1099 enrolled patients were consisted of 243 early RA (disease duration < 6 months) and 856 established RA (disease duration ≥ 6 months) patients. When we further stratified the patients by RA disease duration, the detrimental effect of overlapping SS on RA patients’ therapeutic response was only stably observed in established RA patients, in contrast to be uncertain and statistically insignificant in early RA patients.

**Table 3:** Hazard ratios for reaching remission/low disease activity associated with overlapping SS according to the stratification

	Remission					Remission/LDA			
	DAS28-ESR	DAS28-CRP	SDAI	CDAI	Boolean	DAS28-ESR	DAS28-CRP	SDAI	CDAI
Unmatched cohort									
Age	0.74 (0.65, 0.84)^†^	0.93 (0.84, 1.04)	0.94 (0.82, 1.07)	0.90 (0.78, 1.03)	0.96 (0.84, 1.10)	0.87 (0.79, 0.97)*	0.94 (0.85, 1.03)	0.92 (0.84, 1.01)	0.91 (0.83, 1.00)
RF/ACPA	0.68 (0.62, 0.75)^†^	0.80 (0.74, 0.87)^†^	0.82 (0.74, 0.91)^†^	0.77 (0.70, 0.86)^†^	0.83 (0.75, 0.92)^†^	0.76 (0.70, 0.82)^†^	0.80 (0.74, 0.86)^†^	0.79 (0.73, 0.85)^†^	0.78 (0.73, 0.84)^†^
RA duration	1.45 (0.95, 2.22)	1.45 (0.99, 2.14)	1.18 (0.68, 2.05)	0.88 (0.46, 1.68)	1.13 (0.65, 1.97)	1.44 (1.00, 2.08)	1.48 (1.06, 2.07)	1.39 (0.99, 1.95)	1.40 (1.00, 1.96)
Duration ≥ 6 months	0.67 (0.61, 0.74)^†^	0.80 (0.74, 0.87)^†^	0.82 (0.74, 0.91)^†^	0.77 (0.70, 0.86)^†^	0.83 (0.75, 0.92)^†^	0.76 (0.70, 0.82)^†^	0.80 (0.74, 0.86)^†^	0.79 (0.73, 0.85)^†^	0.78 (0.73, 0.84)^†^
DAS28-CRP at 1st visit	0.64 (0.58, 0.70)^†^	0.76 (0.70, 0.82)^†^	0.76 (0.69, 0.84)^†^	0.71 (0.64, 0.79)^†^	0.77 (0.70, 0.85)^†^	0.73 (0.67, 0.79)^†^	0.78 (0.72, 0.84)^†^	0.77 (0.71, 0.82)^†^	0.76 (0.71, 0.82)^†^
Matched cohort									
Age	0.81 (0.67, 0.98)*	0.85 (0.73, 0.99)*	0.91 (0.74, 1.11)	0.91 (0.74, 1.13)	0.91 (0.74, 1.11)	0.86 (0.73, 1.00)	0.86 (0.75, 0.99)*	0.82 (0.71, 0.95)*	0.83 (0.72, 0.95)*
RF/ACPA	0.71 (0.62, 0.82)^†^	0.74 (0.66, 0.83)^†^	0.82 (0.70, 0.94)*	0.78 (0.67, 0.91)^†^	0.80 (0.69, 0.93)^†^	0.73 (0.65, 0.82)^†^	0.76 (0.68, 0.84)^†^	0.74 (0.66, 0.82)^†^	0.74 (0.66, 0.82)^†^
RA duration	2.51 (1.36, 4.61)^†^	1.93 (1.15, 3.26)*	1.74 (0.81, 3.73)	1.18 (0.51, 2.73)	1.46 (0.70, 3.04)	2.07 (1.26, 3.40)^†^	1.77 (1.14, 2.74)*	1.61 (1.04, 2.47)*	1.64 (1.06, 2.53)*
Duration ≥ 6 months	0.70 (0.61, 0.81)^†^	0.74 (0.66, 0.83)^†^	0.82 (0.71, 0.95)*	0.78 (0.67, 0.92)^†^	0.81 (0.69, 0.94)*	0.73 (0.65, 0.82)^†^	0.76 (0.68, 0.84)^†^	0.74 (0.66, 0.82)^†^	0.74 (0.66, 0.82)^†^
DAS28-CRP at 1st visit	0.70 (0.61, 0.81)^†^	0.73 (0.65, 0.82)^†^	0.78 (0.67, 0.90)^†^	0.75 (0.64, 0.87)^†^	0.77 (0.66, 0.89)^†^	0.71 (0.64, 0.80)^†^	0.73 (0.65, 0.81)^†^	0.71 (0.64, 0.79)^†^	0.71 (0.64, 0.79)^†^
Trimmed cohort									
Age	0.80 (0.66, 0.98)*	0.81 (0.69, 0.95)*	0.90 (0.73, 1.11)	0.87 (0.70, 1.08)	0.90 (0.73, 1.11)	0.83 (0.71, 0.98)*	0.83 (0.72, 0.97)*	0.80 (0.69, 0.93)^†^	0.81 (0.70, 0.94)^†^
RF/ACPA	0.74 (0.64, 0.85)^†^	0.74 (0.66, 0.83)^†^	0.83 (0.72, 0.97)*	0.78 (0.67, 0.91)^†^	0.82 (0.70, 0.95)*	0.74 (0.66, 0.83)^†^	0.75 (0.68, 0.84)^†^	0.74 (0.66, 0.82)^†^	0.74 (0.66, 0.82)*
RA duration	2.49 (1.35, 4.59)^†^	1.91 (1.13, 3.22)*	1.72 (0.80, 3.70)	1.16 (0.50, 2.70)	1.45 (0.69, 3.01)	2.05 (1.25, 3.37)*	1.85 (1.19, 2.87)*	1.68 (1.08, 2.59)*	1.71 (1.10, 2.65)^†^
Duration ≥ 6 months	0.72 (0.63, 0.83)^†^	0.74 (0.65, 0.83)^†^	0.83 (0.72, 0.97)*	0.79 (0.67, 0.92)^†^	0.82 (0.71, 0.96)*	0.74 (0.66, 0.83)^†^	0.75 (0.68, 0.84)^†^	0.73 (0.66, 0.82)^†^	0.73 (0.66, 0.82)^†^
DAS28-CRP at 1st visit	0.71 (0.62, 0.82)^†^	0.72 (0.64, 0.81)^†^	0.79 (0.68, 0.91)^†^	0.74 (0.63, 0.86)^†^	0.77 (0.66, 0.90)^†^	0.71 (0.64, 0.80)^†^	0.72 (0.65, 0.80)^†^	0.71 (0.64, 0.79)^†^	0.71 (0.64, 0.79)^†^

Values are presented as stratified hazard ratio (95% CI) for reaching remission and/or low disease activity based on DAS28-ESR, DAS28-CRP, SDAI, and CDAI in RA patients according to age, RF/ACPA status, RA duration, and DAS28-CRP at 1st visit

*Statistically significant at the level of 0.05

^†^Statistically significant at the level of 0.01

## Discussion

The study based on our follow-up cohort in real medical practice showed that overlapping SS reduced the likelihood of reaching remission or LDA by approximately 20–30% in RA patients, regardless of age, RF/ACPA status, and DAS28-CRP at first visit. Nevertheless, the detrimental effect of overlapping SS on therapeutic response persisted only in established RA patients, instead of early RA patients. The exact reason to explain this finding is unknown. We postulate that the effect of overlapping SS on RA largely depends on the degree of RA therapeutic response itself. It has been generally accepted that early RA patients usually respond better to therapy than established RA, embodied in more to achieve early remission, as well as sustained remission. Therefore, the effect of overlapping SS on reducing RA patients’ therapeutic response becomes relatively weak in early RA patients.

ESR was most often affected by hypergammaglobulinemia associated with overlapping SS. But other individual component of composite RA disease activity score was also inevitably affected by overlapping SS. The impact of overlapping SS on the assessment of RA disease activity was similar regardless of the composite score applied, but SDAI performed the best with least interference by overlapping SS in the matched cohort.

The impact of overlapping SS on the therapeutic response in RA patients is perhaps originated from the crosstalk between the mechanisms of two underlying diseases, which may be explained from four aspects.

### Genetics

It has been estimated that the heritability of RA is approximately 60% [22]. On the one hand, HLA-DRB1*04 allele has been confirmed to confer risk for ACPA-positive RA [23], while on the other hand, HLA-DRB1*03 has been associated with both anti-SSA and anti-SSB production in SS [24]. Apart from classical HLA genes, some genes involved in type I IFN signaling, such as signal transducer and activator of transcription 4 (STAT4), have also been exemplified in the pathogenesis of both RA and SS [25, 26].

### Epigenetics

Epigenetics is currently believed to bridge the gap between environment and genomic DNA in RA [27]. As the most widely studied epigenetic marker, reduced DNA methylation at IFN-induced genes has been observed in RA and SS [28, 29]. MicroRNAs, the endogenous single-stranded non-coding RNAs of approximately 22 nucleotides in length, are considered as negative regulators of immunity [30]. Overexpression of microRNA-146a has been confirmed to be involved in the pathogenesis of RA and SS [31, 32].

### Environment

The role of Epstein-Barr (EB) virus infection has been depicted in the pathogenesis of both RA and SS [33, 34]. Recent studies revealed the interaction between EB nuclear antigen 2 (EBNA2) and genetic loci associated with RA, EBNA-1 and Ro-60 antigen in SS [34, 35]. Besides, a strict relationship between chronic periodontitis and RA and a verified association between chronic periodontitis exposure and SS risk have also been demonstrated [36, 37].

### Adaptive immune system

While the aforementioned genetic and epigenetic association has illustrated the impact of innate immunity, type I IFN signature in particular, the adaptive immunity also bears some resemblance. As we all know, dysregulation of T cells is strongly implicated in the pathogenesis of RA [38]. The pathogenic role of CD4^+^ T cells in the development of SS has been proved, in which aberrant activated T cells facilitate B cell activation forming a positive feedback loop [39]. A recent trial called ROSE aiming to explore the effectiveness of abatacept in RA-SS justifies the crosstalk of T cells between the two diseases [40]. Additionally, B cells are considered as central actors of SS pathogeny, and excess B cell activating factor (BAFF) level is likely to mediate autoreactive B cell accumulation [41], while in the pathogenesis of RA, B cells are involved mainly by producing autoantibodies targeted citrullinated proteins [42]. Previous studies have shown that elevated BAFF levels were positively correlated with RF/ACPA and radiographic progression in RA patients [43–45].

From what has been discussed above concerning the possible crosstalk in the mechanism behind the two diseases, it is reasonable to assume that overlapping SS does harm to therapeutic response and/or outcome in RA patients. Nevertheless, the therapeutic response is always influenced by many factors, making it difficult to determine the effect of overlapping SS, which happens to be settled by PSM. As far as we know, this is the first study to explore the effect of overlapping SS on RA therapeutic response.

The presence of poor prognostic factor (for instance, persistent high disease activity, RF/ACPA positivity) is regarded as critical obstacles to T2T [16]. Most of the cross-sectional studies demonstrated more severe arthritis and worse radiographic outcomes in RA-SS patients compared with RA-noSS patients [5, 11]. Therefore, overlapping SS should be regarded as one of the poor prognostic factors of RA.

There are some limitations of the study based on retrospective cohort of RA in a single center. First, the impact of overlapping SS on the radiographic progress of RA could not be explored due to the unavailability of complete imaging archives. Second, the effect of SS disease activity on the RA management could not be assessed. Third, specific disease modified anti-rheumatic drug was not strictly controlled when correcting confounding effects associated with treatment. There was no pre-defined therapeutic flow in the study, and thus, it was unfeasible to compare the treatment between the RA and RA-SS groups due to hundreds of different drug dosages and combinations. Nevertheless, T2T strategy is key to successful RA management, regardless of any specific drug. Therefore, the results of our study by using “whether under T2T concept” as an indicator variable to control the confounding effect caused by treatment differences are convincing.

## Conclusions

Overlapping SS reduced the probability of reaching RA remission/LDA by approximately 20–30%. Based on the shared mechanisms and observational findings, overlapping SS should be considered as one of the poor prognostic factors of RA. Exploring the effect of overlapping SS on the radiographic outcomes in RA patients is deserved.

## Supplementary information

**Additional file 1: Table S1.** Validation Characteristics in the Unmatched and Propensity-Score Matched Cohorts. **Table S2.** Hazard Ratios for Reaching Remission/Low disease activity in RA patients Associated with Overlapping SS (Before deletion of data at infection episodes). **Table S3.** Hazard Ratios for Remission/Low disease activity Associated with Overlapping SS According to the Stratification of RF or ACPA

## Data Availability

Please contact the author for data requests.

## Abbreviations

SS: Sjogren’s syndrome
RA: Rheumatoid arthritis
DAS28: Disease Activity Score based on 28-Joint Counts
SDAI: Simplified Disease Activity Index
CDAI: Clinical Disease Activity Index
28SJC: 28-swollen joint count
28TJC: 28-tender joint count
PtGA: Patient global assessment of disease activity
VAS: Visual analog scale
EGA: Evaluator’s global assessment of disease activity
ESR: Erythrocyte sedimentation rate
CRP: C-reactive protein
LDA: Low disease activity
RA-SS: Overlapping SS with RA
T2T: Treat-to-target
RF: Rheumatoid factor
ACPA: Anti-citrullinated peptide antibody
PSM: Propensity score matching
ACR: American College of Rheumatology
EULAR: ACR/European League Against Rheumatism
HRs: Hazard ratios
CIs: Confidence intervals
IQR: Interquartile ranges
STAT4: Signal transducer and activator of transcription 4
EBNA2: EB nuclear antigen 2
BAFF: B cell activating factor
RANKL: Receptor activator of nuclear factor κB ligand
