# Supplementary material for: Overlapping Sjogren’s syndrome reduces the probability of reaching target in rheumatoid arthritis patients: a propensity score matched real-world cohort from 2009 to 2019
Source: Arthritis Res Ther. 2020 May 1;22:100. doi: 10.1186/s13075-020-02189-w (PMC7195774; doi:10.1186/s13075-020-02189-w)
Supplement: Supplementary file 1 — Additional file 1: Table S1. Validation Characteristics in the Unmatched and Propensity-Score Matched Cohorts. Table S2. Hazard Ratios for Reaching Remission/Low disease activity in RA patients Associated with Overlapping SS (Before deletion of data at infection episodes). Table S3. Hazard Ratios for Remission/Low disease activity Associated with Overlapping SS According to the Stratification of RF or ACPA [file 13075_2020_2189_MOESM1_ESM.docx]

| Table S1 Validation Characteristics in the Unmatched and Propensity-Score Matched Cohorts | | | | | | | | |  |  |
| --- | --- | --- | --- | --- | --- | --- | --- | --- | --- | --- |
|  | | | Unmatched Cohort | | |  | Matched Cohort | | | |
|  | | | RA with SS | RA without SS | |  | RA with SS | | RA without SS | |
|  | | | (n=129) | (n=970) | |  | (n=126) | | (n=126) | |
| Positive for RF, n (%) | | | 116 (89.9) | 724 (74.6) | |  | 115 (91.3) | | 105 (83.3) | |
| Positive for ACPA, n (%) | | | 117 (90.7) | 773 (79.7) | |  | 116 (92.1) | | 114 (90.5) | |
| Validation by anti-SSA, n (%) | | | 100 (77.5) | - | |  | 99 (78.6) | | - | |
| Validation by MSGB, n (%) | | | 29 (22.5) | - | |  | 27 (21.4) | | - | |
| Values are presented as n (%). MSGB refers to minor salivary gland biopsy.  Table S2 Hazard Ratios for Reaching Remission/Low disease activity in RA patients Associated with Overlapping SS (Before deletion of data at infection episodes) | | | | | | | | |  |  |
|  |  |  |  |  |  |  |  |  |  |  |
|  | Unmatched cohort | | | Matched cohort | | | Trimmed cohort | |  |  |
| Remission |  | | |  | | |  | |  |  |
| DAS28-ESR | 0.68 (0.62, 0.75) | | | 0.72 (0.62, 0.82) | | | 0.73 (0.64, 0.85) | |  |  |
| DAS28-CRP | 0.80 (0.74, 0.87) | | | 0.74 (0.66, 0.83) | | | 0.74 (0.66, 0.83) | |  |  |
| SDAI | 0.82 (0.75, 0.91) | | | 0.82 (0.71, 0.95) | | | 0.83 (0.72, 0.97)* | |  |  |
| CDAI | 0.77 (0.70, 0.86) | | | 0.78 (0.67, 0.91) | | | 0.79 (0.67, 0.92) | |  |  |
| Boolean | 0.83 (0.75, 0.92) | | | 0.80 (0.69, 0.93) | | | 0.82 (0.70, 0.95)* | |  |  |
| Remission/LDA |  | | |  | | |  | |  |  |
| DAS28-ESR | 0.76 (0.70, 0.82) | | | 0.73 (0.65, 0.82) | | | 0.74 (0.66, 0.83) | |  |  |
| DAS28-CRP | 0.80 (0.74, 0.86) | | | 0.76 (0.68, 0.84) | | | 0.76 (0.68, 0.85) | |  |  |
| SDAI | 0.79 (0.73, 0.85) | | | 0.74 (0.66, 0.82) | | | 0.74 (0.66, 0.82) | |  |  |
| CDAI | 0.78 (0.73, 0.84) | | | 0.74 (0.67, 0.82) | | | 0.74 (0.67, 0.82) | |  |  |

Values are presented as Hazard Ratio (95% CI) for reaching remission and/or low disease activity based on DAS28-ESR, DAS28-CRP, SDAI, CDAI and main components in RA patients. Unmatched cohort refers to whole sample (n=1099), matched cohort refers to propensity score matched (PSM) patients (n=252) after correcting gender, age, RA duration, RF/ACPA status, DAS28-CRP at 1^st^ visit and T2T or not, trimmed cohort refers to cohort with either trimmed at the 5^th^-95^th^ percentiles of the PSM (n=242). ULN of ESR refers to 15mm/h for male and 20mm/h for female. * refers to statistically significant at the level of 0.05, and other HRs are statistically significant at the level of 0.01.

| Table S3 Hazard Ratios for Remission/Low disease activity Associated with Overlapping SS According to  the Stratification of RF or ACPA | | | | | | | | |
| --- | --- | --- | --- | --- | --- | --- | --- | --- |
|  | Unmatched cohort | |  | Matched cohort | |  | Trimmed cohort | |
|  | RF | ACPA |  | RF | ACPA |  | RF | ACPA |
| Remission |  |  |  |  |  |  |  |  |
| DAS28-ESR | 0.71 (0.64, 0.78) | 0.67 (0.61, 0.74) |  | 0.72 (0.62, 0.82) | 0.70 (0.60, 0.81) |  | 0.74 (0.64, 0.85) | 0.72 (0.63, 0.84) |
| DAS28-CRP | 0.83 (0.76, 0.90) | 0.80 (0.73, 0.87) |  | 0.74 (0.66, 0.83) | 0.73 (0.65, 0.83) |  | 0.74 (0.66, 0.83) | 0.73 (0.65, 0.83) |
| SDAI | 0.85 (0.77, 0.94) | 0.81 (0.73, 0.89) |  | 0.82 (0.71, 0.95) | 0.82 (0.70, 0.95) |  | 0.84 (0.72, 0.97)* | 0.83 (0.72, 0.97)* |
| CDAI | 0.81 (0.73, 0.89) | 0.77 (0.69, 0.85) |  | 0.79 (0.67, 0.92) | 0.78 (0.67, 0.92) |  | 0.79 (0.67, 0.92) | 0.79 (0.67, 0.93) |
| Boolean | 0.87 (0.78, 0.96) | 0.82 (0.74, 0.90) |  | 0.81 (0.69, 0.94) | 0.80 (0.69, 0.94) |  | 0.82 (0.71, 0.96)* | 0.82 (0.70, 0.96)* |
| Remission/LDA |  |  |  |  |  |  |  |  |
| DAS28-ESR | 0.79 (0.73, 0.85) | 0.75 (0.69, 0.82) |  | 0.74 (0.66, 0.83) | 0.71 (0.63, 0.80) |  | 0.75 (0.66, 0.84) | 0.73 (0.64, 0.82) |
| DAS28-CRP | 0.82 (0.76, 0.88) | 0.79 (0.74, 0.86) |  | 0.76 (0.69, 0.85) | 0.74 (0.67, 0.83) |  | 0.76 (0.68, 0.85) | 0.74 (0.67, 0.83) |
| SDAI | 0.81 (0.75, 0.87) | 0.78 (0.72, 0.84) |  | 0.74 (0.67, 0.82) | 0.72 (0.64, 0.80) |  | 0.74 (0.66, 0.82) | 0.72 (0.65, 0.81) |
| CDAI | 0.81 (0.75, 0.87) | 0.77 (0.72, 0.83) |  | 0.74 (0.67, 0.82) | 0.72 (0.65, 0.80) |  | 0.74 (0.67, 0.82) | 0.72 (0.65, 0.81) |

Values are presented as stratified Hazard Ratio (95% CI) for reaching remission and/or low disease activity based on RF or ACPA status. * refers to statistically significant at the level of 0.05, and other HRs are statistically significant at the level of 0.01.
